# A Challenging Diagnosis of Kawasaki Disease Shock Syndrome Complicated by Bilateral Pleural Effusion: A Case Report and Literature Review

**DOI:** 10.7759/cureus.49671

**Published:** 2023-11-29

**Authors:** Linah Saleh Abbas Alghamdi, Ali Yahya B Alzahrani, Fahad A Alghamdi, Saleh J ALghamdi

**Affiliations:** 1 Pediatrics and Child Health, King Fahad General Hospital, Al Baha, SAU; 2 Radiology, King Fahad General Hospital, Jeddah, SAU

**Keywords:** auto-immune disease, medium vessel vasculitis, bilateral pleural effusion, kawasaki disease shock syndrome, kawasaki disease (kd)

## Abstract

Kawasaki disease (KD) is an acute illness primarily affecting children under the age of five. It is characterized by fever and inflammation of small to medium-sized arteries. This case report presents the case of a nine-year-old boy with KD who developed Kawasaki disease shock syndrome (KDSS) complicated by bilateral pleural effusion, which is a rare occurrence. KDSS is defined as KD accompanied by low blood pressure or signs of inadequate blood flow, leading to increased cardiovascular complications. The patient exhibited typical KD symptoms, including conjunctivitis, mucosal changes, rash, extremity swelling, and lymphadenopathy. Additionally, he presented with shock symptoms, such as hypotension and tachycardia. Laboratory findings showed elevated inflammatory markers. Prompt diagnosis and treatment are crucial to prevent coronary artery lesions and other severe complications. The patient received intravenous immunoglobulin and showed significant improvement, with resolution of fever and respiratory distress. Follow-up echocardiography revealed normal results. While pulmonary involvement in KD is rare, the presence of bilateral pleural effusion underscores the challenges in diagnosing KDSS. Early recognition and management are essential for favorable outcomes in KD and its complications.

## Introduction

Kawasaki disease (KD) represents an acute pathological condition with an abrupt and intense onset, primarily exhibiting its effects on the pediatric demographic, particularly those within the tender age group of infancy to five years. It is characterized by a sudden fever and inflammation affecting arteries of varying sizes, ranging from small to medium-sized [1]. Initially, KD was believed to be a variant of mucocutaneous-ocular syndrome until it was reported by Dr. Tomisaku Kawasaki as a distinct set of fifty cases, leading to its identification as KD. There have been indications proposing that the inflammatory response in KD could potentially be initiated by undisclosed infections, particularly in genetically susceptible individuals [1,2]. The diagnosis of KD involves identifying the presence of a persistent febrile state persisting for a minimum duration of five days, concomitant with the presence of no less than four characteristic manifestations from the following diagnostic criteria: (I) non-purulent conjunctivitis bilaterally exhibiting sparing of the limbal region, (II) oropharyngeal mucosal changes encompassing erythematous fissured lips, a tongue displaying a strawberry-like appearance, or diffuse erythema within the oropharynx, (III) a generalized rash of indeterminate nature, (IV) edema and erythema affecting the extremities, and (V) unilateral cervical lymphadenopathy. In the cases where criteria are met, a diagnosis of KD is considered [3,4]. Timely identification and treatment of KD are crucial to minimize the vulnerability of coronary artery affections, which have the potential to lead to myocardial infarction and fatality [2]. During the acute phase of KD, experiencing hemodynamic instability rarely happens.In 2009, Kanegaye et al. introduced the term KD shock syndrome (KDSS) to describe this condition [3]. The specific etiology of profound hypotension in KDSS is still not fully understood, but it is hypothesized to be connected to ongoing vasculitis, leakage of capillaries, and impaired myocardial function, and dysregulation of cytokines at a systemic level. KDSS, further complicated by the presence of bilateral pleural effusion, is extremely rare, with only one reported case published in the literature [5].

## Case presentation

An eight-year-old boy was brought to the hospital due to a sore throat and fever accompanied by headaches that had persisted for five days. The child's parents mentioned that he had been in good health until a week ago when he developed a high-grade and almost continuous fever, which was only mildly relieved by paracetamol. The child was brought to our hospital's emergency department (ED), where an examination revealed the presence of bilateral non-purulent conjunctivitis, cracked lips, a red tongue resembling a strawberry, and a congested throat with red and swollen tonsils. The child exhibited signs of shock, including a prolonged capillary refill time (CRT) of more than four seconds, strong central pulses, weak peripheral pulses, hypotension, and tachycardia. During the initial assessment in the ED, the patient's blood pressure was measured at 80/65 mmHg, temperature at 38.5 degrees Celsius, oxygen saturation at 97%, and pulse rate at 168 beats per minute. To address the condition, two boluses of fluid (20 ml/kg) were administered rapidly, blood investigations were conducted, and upon suspicion of septic shock, the primary physician initiated treatment with intravenous ceftriaxone. Subsequently, the child was transferred to the pediatric intensive care unit (PICU) for meticulous surveillance and monitoring. Table 1 presents the noteworthy results of the comprehensive blood tests conducted, providing valuable insights into the patient's condition.

**Table 1:** Blood test results at presentation, third day and fifth day of admission

Blood investigation	At presentation	Third day	Fifth day
Hemoglobin	11.3 g/dl	9.2 g/dl	13.4 g/dl
White blood cells	10.7 X 109		
Neutrophil	91.8 %		
Lymphocyte	2.40%		
Platelet	254 X 109	663 X 109	1123 X 109
Serum aspartate transaminase	360 U/L		
Serum alanine transaminase	199 U/L		
Albumin	28.3 g/l	25.1 g/l	
Prothrombin time	23.30 sec		
Activated partial thromboplastin time	46.5 sec		
International normalized ratio	1.8 mg/l		
Erythrocytes sedimentation rate	100 MM/HR	150 MM/HR	125 MM/HR
C-reactive protein	2.35 mg/dl		
Lactic acid	14.8 mmol/l	7.8 mmol/l	1.7 mmol/l

**Figure 1:**
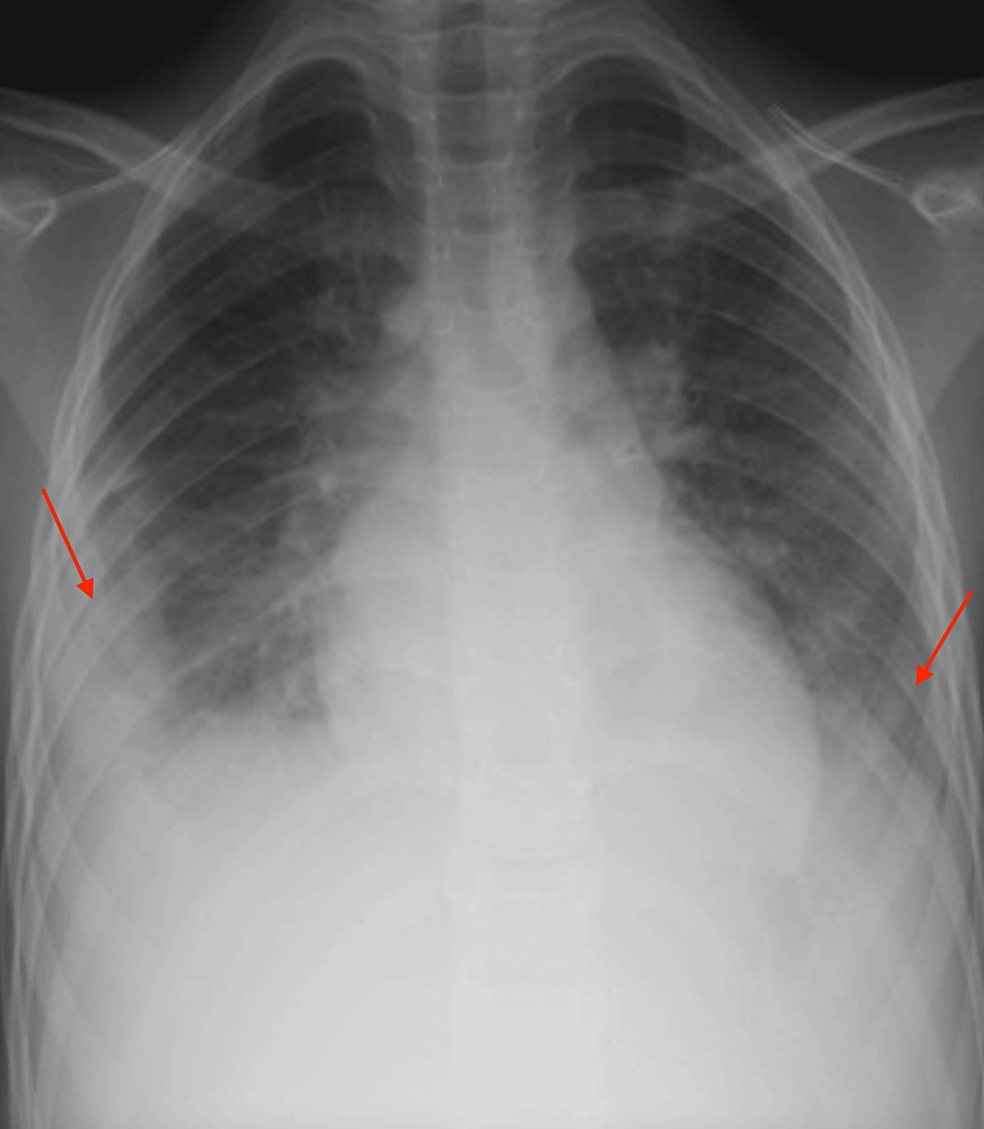
The initial presentation of the patient reveals bilateral pleural effusion, with a greater extent observed on the right side, accompanied by obliteration of the costophrenic angles

On the third day after admission, the laboratory results indicated a decreased albumin level of 25.1 g/l. To improve this, a 20% albumin transfusion was administered at a dosage of one gram per kilogram of the child's weight. Additionally, the initial presentation of the patient revealed bilateral pleural effusion, with a greater extent observed on the right side, accompanied by obliteration of the costophrenic angles. No evidence of consolidation was noted (Figure 1).

**Figure 2:**
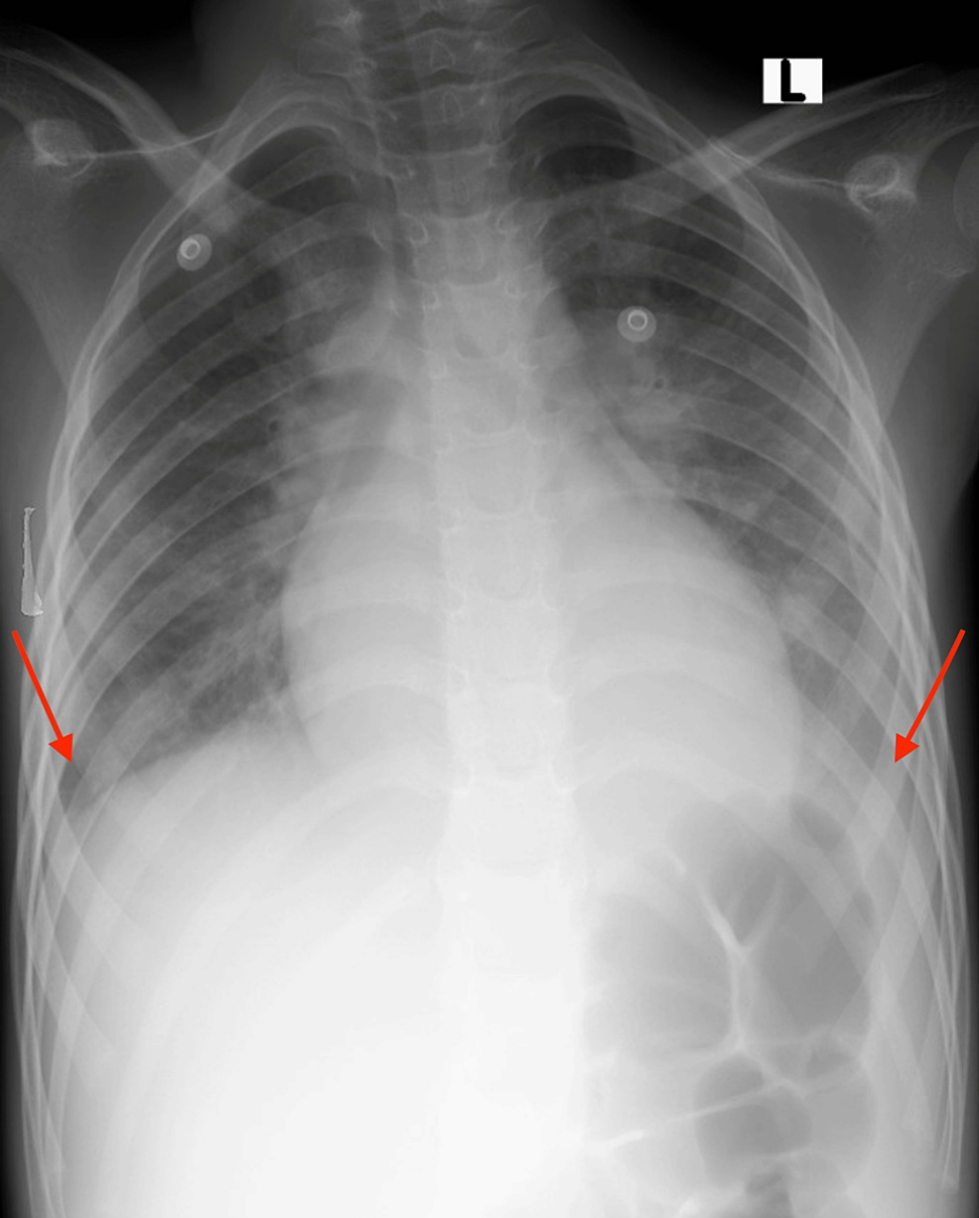
Follow-up images were captured seven days after the presentation and two days into the IVIG course, demonstrating significant improvement in the previously detected pleural effusion IVIG - intravenous immunoglobulin

Furthermore, an ultrasound examination of the abdomen and chest was conducted, which identified mild bilateral pleural effusion and mild free fluid in the pelvic region. After stabilizing for five days and receiving intravenous antibiotic treatment, the child continued to experience a persistent high-spiking fever. Additionally, generalized maculopapular rashes were observed on the child's body, accompanied by peeling of the hands and feet, indicative of itchy skin rashes. An echocardiography was performed that revealed mild left ventricular dilatation with preserved function and mild mitral regurgitation. Given the combination of elevated inflammatory markers, features of KD, and evidence of hypoperfusion, the patient was diagnosed with KDSS. To address this condition, intravenous immunoglobulin (IVIG) was administered at a dosage of two grams per kilogram. Additionally, acetylsalicylic acid (100 mg/kg/day) was initiated, and careful monitoring of the fever was implemented as part of the management plan. The treatment strategy aimed to mitigate the effects of KDSS and promote the patient's recovery while closely monitoring clinical progress.The patient experienced a significant improvement in the condition, with the fever and respiratory distress resolving rapidly. Subsequent chest X-rays showed a gradual resolution of the pleural effusion, indicating a positive response to treatment (Figure 2).

To monitor the possibility of cardiac complications associated with KD, the patient was scheduled for follow-up visits at two weeks and six weeks. Fortunately, echocardiography performed during these visits revealed normal results, indicating the absence of cardiac issues. As a result, the use of acetylsalicylic acid was discontinued after six weeks since the echocardiography findings remained normal. Although KD is a systemic vasculitis that can affect multiple organs, significant pulmonary involvement is uncommon. Early detection of KDSS, a severe form of KD characterized by shock and organ dysfunction, can be challenging.

In summary, the patient's condition improved dramatically, with the resolution of fever and respiratory distress. Follow-up echocardiography revealed normal results, leading to the cessation of acetylsalicylic acid medication. While KD can involve various organs, pulmonary involvement is typically rare, and the early diagnosis of KDSS poses difficulties.

## Discussion

Dr. Tomisaku Kawasaki, a Japanese physician, was the first to describe KD [1,2]. KD is characterized by a medium-sized vessel vasculitis that impacts multiple organ systems throughout the body [3]. The incidence of KD is higher in children from Asia compared to the Western world. Although several theories, including the hypothesis of infectious etiology, theory of vaccines exposure, and immune factor dysregulation, have been proposed, the precise etiology of KD remains unidentified [4,6]. However, it is believed that an unknown trigger leads to an inflammatory response, activating both the innate and adaptive immune systems and causing the release of various inflammatory cytokines that affect the walls of the systemic arteries [5,7-8]. The diagnosis of KD in this case was made using the criteria established by the American Heart Association [6]. Kawasaki disease (KD) can exhibit atypical symptoms that encompass various systems, including the pulmonary system. Although pulmonary involvement in KD is infrequent, it can manifest as pneumonia, pulmonary nodules, bronchopneumonia, hydro-pneumothorax, and pleural effusion. These uncommon pulmonary manifestations underscore the diverse clinical presentation of KD and emphasize the importance of considering such manifestations when diagnosing and managing the disease [5,8,9]. Patients with pulmonary involvement may have a higher risk of coronary artery abnormalities due to delays in diagnosing KD and administering IVIG treatment [3-5]. Among the various complications observed in KD, cardiac manifestations, including coronary artery aneurysms, myocarditis, myocardial infarction, and sudden cardiac death, are the most prevalent and grave. Nonetheless, KD can also present in the form of KDSS [10]. KDSS, as described by Kanegaye et al. in 2009, is characterized by the simultaneous presence of KD symptoms and specific indicators, such as systolic hypotension (systolic blood pressure below -2 standard deviations relative to sex and age) accompanied by a decline of over 20% from baseline blood pressure, or signs suggestive of inadequate tissue perfusion[3]. It is crucial to be cognizant of the potential occurrence of KDSS as it represents a distinct clinical entity within the spectrum of KD, necessitating prompt recognition and management to ensure optimal patient outcomes [10].

Although the specific etiology of profound hypotension in KDSS is still not fully understood, it is hypothesized to be connected to ongoing vasculitis, leakage of capillaries, impaired myocardial function, and dysregulation of cytokines at a systemic level, resulting in cardiogenic and/or distributive shock [6]. In the pediatric population, when children with shock or hypotension require admission to the intensive care unit and fail to respond to antibiotic therapy, it is crucial to consider the possibility of KDSS, even if they do not initially fulfill the diagnostic criteria for KD and exhibit negative blood and urine cultures. KDSS should be contemplated as a potential underlying condition in such cases, highlighting the significance of maintaining a high index of suspicion and employing a comprehensive diagnostic approach that extends beyond traditional criteria and culture results. Timely recognition and appropriate management of KDSS are paramount to optimize patient outcomes in these challenging clinical scenarios [6-9].

In our case study, an eight-year-old boy without any prior medical conditions exhibited persistent fever, redness and swelling of the lower limbs, redness in both eyes, unresponsive shock, low levels of albumin, significantly increased platelet count and elevated levels of C-reactive protein (CRP) and erythrocyte sedimentation rate (ESR). Despite the presence of these clinical indicators, the diagnosis of KDSS was not definitively confirmed until a subsequent phase of the illness. The confirmation of KDSS, in this particular case, was prompted by the observation of desquamation (skin peeling) on the hands and feet, an uncommon yet severe complication of KD. This case serves as a notable example, highlighting the significance of promptly recognizing and remaining vigilant for the manifestation of KDSS. Early detection is essential as it enables timely initiation of treatment, which can have a profound impact on patient outcomes. To the best of our knowledge, this represents only the second documented case of KDSS with pleural effusion reported globally. This underscores the rarity of such occurrences and emphasizes the importance of further research and continued awareness of this complex clinical entity.

Literature review

A literature review was conducted using PubMed with the search terms "(Kawasaki disease shock syndrome OR Kawasaki disease shock syndrome) AND (pleural effusion)" to identify relevant articles. The search yielded a total of five articles. After careful evaluation, four articles were excluded based on the following criteria: three of them focused on KD or multisystem inflammatory syndrome rather than KDSS, and one article specifically examined the adult age group. The remaining article provided valuable insights into the topic at hand and presented a case that closely resembled the report being discussed in this article. The findings from this article contribute to the understanding and documentation of KDSS with pleural effusion. The selected article, authored by Natterer et al. [5], provides a detailed description of a similar case involving a patient with KDSS and pleural effusion. The case presentation, clinical features, diagnostic approach, and treatment modalities were discussed in depth, highlighting the importance of early recognition and management of this rare complication. The findings from this case study align with the observations made in our report. Overall, the literature review revealed one relevant article that significantly contributes to the existing knowledge on KDSS with pleural effusion. The inclusion of this case study enhances the scientific basis and clinical relevance of our research, further emphasizing the importance of recognizing and managing this unique presentation of KDSS. 

## Conclusions

KD is a condition affecting medium-sized blood vessels, and it typically follows a mild course if detected and treated early. It's crucial to be aware that fever is the most common symptom in children, and a wide range of conditions, including vasculitis, should be considered to avoid overlooking KD. Therefore, clinicians should maintain a high index of suspicion for KD and consider pulmonary involvement as a potential complication. In summary, this case highlights the importance of considering KDSS as a differential diagnosis in children presenting with prolonged fever and shock. Early recognition, timely treatment, and close monitoring are key to preventing severe complications and ensuring favorable outcomes in patients with KD and its associated syndromes. Further research and awareness are needed to better understand the underlying mechanisms of KD, its varied presentations, and the optimal management strategies. By expanding our knowledge and improving diagnostic capabilities, we can enhance the care provided to children affected by this challenging disease.
